# “For the services of shipwrights, coopers, and grumettas”: Freetown’s ship repair cluster in nineteenth-century Sierra Leone

**DOI:** 10.1177/0073275320945117

**Published:** 2020-08-13

**Authors:** Bronwen Everill

**Affiliations:** Gonville & Caius College, University of Cambridge, UK

**Keywords:** Shipbuilding, West Africa, technology transfer, maritime economy, port specialization, repair, cluster, Sierra Leone

## Abstract

This article looks at the development of Sierra Leone’s ship repair cluster, particularly focusing on the period 1780 to 1860. It argues that several factors contributed to the colony’s ability to develop a ship repair cluster. The first was the local environment, which provided both a safe harbor for ships and boats, and local materials that could be used on European and American ships. Secondly, the port’s increasing commercial role and its unique position as the site of the Courts of Mixed Commission for the adjudication of condemned slaving ships after the abolition of the slave trade gave ship’s carpenters access to a wide and varied range of both customers and supplies. Finally, these material effects were enhanced by the cluster’s effect on knowledge spillover and on-the-spot tacit knowledge creation as disruptions in the supply chain, competition with slave traders, and other local circumstances fostered innovation in Freetown’s repair cluster.

## Introduction

In 1831, the British colonial government in Freetown, Sierra Leone, in West Africa, undertook the first comprehensive census of its population. Freetown, founded in the late eighteenth century as an antislavery colony, had a diverse population comprised of black settlers from North America and Jamaica, migrants from the region around the West African colony, and Africans liberated from slave trading vessels and resettled in the colony after the British abolition of the slave trade in 1808. The census showed that, concentrated in several streets near the harbor, in under a square mile, the colony was also home to eighteen ship’s carpenters, one sailmaker, two boatbuilders, nine ropemakers, nine blacksmiths, a caulker, merchants selling the materials necessary for ship repairs, and brokers offering shipping insurance.^1^

This article looks at the development of Freetown’s cluster of ship repair facilities, particularly focusing on the period 1780 to 1860. The establishment of this anti-slave trading colony at the mouth of the Sierra Leone River in 1787 helps to reveal how and why a port city might develop a reputation for ship repair. The supporters of the colony hoped to encourage the growth of “legitimate commerce” in primary products to attract African producers away from slave trading. To compete with slave traders, Freetown’s merchants and Sierra Leone’s colonial government pursued a range of strategies to make trade in the colony attractive. Providing the material, the skilled labor, and the financing to undertake ship repairs enabled the colony’s government and its increasingly diverse group of residents to begin to overtake their commercial rivals on the coast, and in so doing make a case for the profitability of abandoning the slave trade for legitimate commerce.

This article argues that several factors contributed to the colony’s ability to develop a ship repair cluster. Scholars of knowledge exchange have looked at the development of industry “clusters” to understand how geography contributes to the development of knowledge and expertise. A cluster is a “geographic concentration of interconnected companies and institutions in a particular field” at different levels of the supply chain.^2^ In the case of Sierra Leone, the cluster was facilitated by several factors. The first was the local environment, which, as the largest natural harbor in West Africa, provided both a safe harbor for ships and boats, and local materials that could be used on European and American ships. Secondly, the port’s increasing commercial role and its unique position as the site of the Courts of Mixed Commission for seizing and condemning slaving ships after the abolition of the slave trade gave ship’s carpenters access to a wide and varied range of customers, supplies, and labor. Finally, these material effects were enhanced by the consequences of the cluster’s knowledge spillover and on-the-spot tacit knowledge creation, as disruptions in the supply chain, competition with slave traders, and other local circumstances fostered financial innovation in Freetown’s repair cluster. The unique features of Freetown set it apart from its competitors in the region, but these features also highlight how knowledge, materials, institutions, and technological and financial innovations could work more generally to generate complementary industries in ways that could be comparable to other nineteenth-century colonial port cities.

## Sierra Leone’s institutional context

Sierra Leone is an interesting case for investigating the development of hubs of transportation repair. British philanthropists established Freetown as an antislavery colony both to offer a new start to black Britons from around the empire and to offer a commercial and political alternative to the slave trade. Located between the slave trading hotspots of the Rio Pongo and Rio Nunez to the north and Sherbro and Gallinas to the south, the colony was meant to provide a new life for formerly enslaved Africans and people of African descent, to offer alternative forms of commerce to reduce Africans’ reliance on the slave trade for income, and to deter slave traders. From 1808, when the Sierra Leone Company transferred the colony to the British government, Freetown was a base for the British Anti-Slave Trade Naval Squadron and the seat of a court for the adjudication of their illegal slave ship captures.^3^ In order to attract “legitimate” commerce and build a robust, non-slave trading economy, however, the Freetown colonial government needed to make the port attractive for both regional and global trade. One way that the colony’s settlers and government did this was through Freetown’s ship repair cluster.

Once the British government legally abolished its participation in the Atlantic slave trade in 1807, slave ships caught by the British West African naval patrol were adjudicated at the Vice-Admiralty Courts in Freetown. After 1817, the new Courts of Mixed Commission replaced the wartime Vice-Admiralty Court, and the British collaborated with the Portuguese, Dutch, and Spanish in prosecuting illegal slaving off the coast of West Africa. When a ship was captured, it was taken to Freetown where a court determined whether a ship was in fact in violation of the ban on slave trading. Then the enslaved captives were freed and apprenticed, and the ships were auctioned to individuals, or to groups of Sierra Leoneans who raised money jointly to buy the condemned ships or their parts for coastal trading.^4^

Because of the Courts of Mixed Commission, supplies of imported materials needed for ship repair – particularly sails and copper – were more likely to be found in Sierra Leone as the nineteenth century progressed, and the rapid expansion of the population through the resettlement of the “Liberated Africans” created the conditions for a growing maritime service sector. The environmental reputation of the region was enhanced by the advantages in materials and personnel conferred by the institution of the Mixed Commission Courts.

The project of the British Sierra Leone colony to eliminate the slave trade saw the merchants take advantage of the local environment, as well as Freetown’s political and human capital, in ways that fostered the development of an industry cluster, which led to further innovations and (some) success in their goal. Sierra Leone’s maritime economy – well-documented because of the region’s particular history – presents a window into the kinds of technical, human, environmental, and material resources that suited a nineteenth-century port for global ship and boat repair.

## Sierra Leone’s environmental advantage

Freetown harbor was one of only a few natural harbors on the western coast of Africa. This harbor and the surrounding rivers facilitated crucial ship repair by providing protection against the open seas when conducting repairs to masts, sails, and fittings, and enabling captains to bring ships ashore for repairs of the hull, as described in various contemporary mariners’ guides.^5^ The French slave trader, Jean Barbot, who wrote an influential account of the Upper Guinea Coast published in English in the early eighteenth century, reported of the Rio Pongo, north of Sierra Leone, that

Many *European* ships visit this river, as well on account of trade, as for its convenient situation, for the cleaning and refitting of vessels: those who go on the last account, unlade their anchors, guns, water-casks, and other like heavy or bulky carriage, on the *Prince’s* island of *Pongo*, and by the help of the flood, get their ships as far on land as is possible, that by means of the strong ebb, they may be on a sort of dry ground, and thus more easily repair their vessel.^6^

Historian Colleen Kriger notes, “the Sierra Leone estuary, where there were innumerable sheltered bays and inlets for establishing makeshift shipyards, had long been a choice site for hauling company vessels ashore for repairs.”^7^ This reputation as a good stopping point for repairs preceded the antislavery colony’s establishment, and offered something for colonial governors and settlers to build on, if they could monopolize the rivers and materials used by slave traders. The existing environmental suitability for ship repairs was incorporated into a concerted attempt by Freetown’s government to control access to that environment.

In 1797, the Governor of Sierra Leone recounted an incident that shows how a suitable environment could help an area in developing a ship repair-oriented economy. In this incident:

A Settler came to me today with a complaint against the natives living in Fora Bay. He had gone up the creek with his trading boat and. . .taken it ashore at a convenient place to have it repaired. He was on the point of launching again and coming round with his vessel when these natives came demanding customs for having laid it ashore on the king’s land and as he refused to pay their demand they took possession of his sails and rudder and carried them to their town.^8^

The residents of Fourah Bay had established a regular system of charging for boats that were brought ashore to be repaired because of the area’s easy access and reputation for good local materials; they did not want to be denied the standard charge for these (admittedly passive) services. But the Sierra Leone government, who were trying to undermine the slave trade in the region, were hoping to expand their territorial reach in order to make it more difficult for slave traders to operate in the region. Incidents like this one typically resulted in the negotiation of new treaties that encouraged favorable treatment of the antislavery colony’s commerce at the expense of their competitors in the slave trade. The colony expanded its reach into local rivers that had longstanding reputations for suitable beaches and materials for repairing European vessels. An article in the *Sierra Leone Gazette* illustrated the success of this approach, noting, “the slave traders and chiefs in the Rio Pongos. . .promise to renounce all traffic in slaves.” The editors were not sure if this grew out of “a feeling of interest in being allowed to trade with this colony” but pointed out that the motivation “is of little consequence, provided the same leads to a cessation of the traffic in human flesh in that fine river.”^9^

As early as 1795, the colony established a factory for trade at Freeport, in the Rio Pongo. This drew the ire of the slave traders there because, although the Freeport factory would be trading for timber and other produce, not slaves, the slave traders rightly worried that the colony would soon monopolize the region’s trade.^10^ By the late 1810s, Freetown’s governor, Charles MacCarthy, signed a treaty with Mongo Dimba, King of the Bagas,

. . .for the use and on the behalf of His Majesty the King of Great Britain, and Ireland; his Heirs, and Successors, the full, and entire free, and unlimited possession and sovereignty of the said Isles de Loss, viz. Factory, Crawford, Tammera, White, and Coral Islands, together with all and every Right and Title to the Navigation, Anchorage, Waterage, Fishing, and other Revenue and Maritime claims, in and over all the Harbours, Bays, Creeks, Roads, Roadsted, and Waters, in and round the islands.^11^

Freetown expanded its jurisdiction, bringing the hotspots of private slave trading within its governance remit in order to both attempt to quash the rampant illegal slave trade with which they were competing and to profit from the reputation of the Isles de Los, the Rio Pongo, Fourah Bay, and the Sierra Leone River as good places to stop off to replenish stocks and repair vessels.

## Local knowledge

While Freetown was increasingly likely to have ship repair materials on hand after 1807, the sporadic nature of all early nineteenth-century supply chains – and especially one that relied on the capture of illegal slaving ships – made the ability to improvise important to the skillset of Africa’s ship’s carpenters.^12^ Boatbuilding was an existing and fairly widespread industry in West Africa, and adaptation and technological responsiveness, rather than stasis, characterized the industry.^13^ With the expansion of Atlantic-oriented trade (and especially the slave trade) from the early seventeenth century, Kevin Dawson explains that existing canoe design was adapted over time to reflect the shift toward cargo loading and surf-ports from earlier lagoon and riverine use. African surf-canoes were adapted with “wash strakes, weatherboards, or splashboards” to increase the space between the water’s surface and the gunwale of the canoe to facilitate the movement of goods and people between ship and shore.^14^ The adaptations further embedded canoe technology as necessary to the commerce of the coast.^15^ Austen and Headrick comment that in addition to adapting canoes for increased coastal trade, “African artisans were able to construct a large number of forty to sixty ton schooners for river and coastal navigation.”^16^ West African canoe-makers thus responded to both changing technological needs and shifting commercial patterns. This history of technological adaptation to facilitate commercial growth echoes the pattern elsewhere globally, where the combination of local and imported technologies in India or the Caribbean, and the important role of commercial mediators, has also been recognized by historians.^17^

While the study of canoe construction in West Africa demonstrates the skills and environmental expertise of local craftsmen, and the importance of local technologies in the expansion of European and West African commercial interactions, European and American oceanic ships arriving in West Africa required not only skilled craftsmen, but skilled craftsmen who could adapt a combination of local and imported materials to the problem at hand. Tacit knowledge might mean knowing what local material could substitute for caulk or rope in the absence of the European import.

Places like the Isles de Los, Rio Pongo, and Fourah Bay had both the environmental requirements for conducting repairs and the local materials. Both Barbot and Álvares commented on the fact that “Forests along the shores of Port Loko Creek and the Rokelle River” near Freetown “offered shipwrights a wealth of raw materials for building, fitting, and repairing coasting and perhaps even maritime vessels. . .Oakum, used for caulking seams of boat hulls, was made locally by processing the bark of another species of tree,” all of which made this region important for the ‘interlopers’ and private traders.^18^ African craftsmen could point crews to plants that were used to make ropes or caulk, provide locally made cotton cloth for sail repairs, or identify which timber would be suitable for masts and for replacing planks.

Ship timber was of crucial importance to Sierra Leone’s reputation for ship repairs and for commerce. Local timber was the most important non-slave export in the early nineteenth century, and the wood was widely touted as strong and of mahogany quality.^19^ Timber factories and sawmills were set up in Port Loko Creek, Bunce Island, Tasso Island, and in the Northern Rivers (Nunez and Pongo) not long after the legal abolition of the slave trade. Competition among merchants for access to the timber trade was the source of regular government correspondence.^20^ The prevalence of sawyers among the occupations listed in the censuses of Freetown and surrounding villages in 1831 was second only to agriculture, highlighting their importance – and the timber industry’s – to the colony’s economy.^21^

Importantly, Sierra Leone’s timber was exported for use in shipbuilding in Britain itself, and so could be trusted for repairs in Sierra Leone. As the *Sierra Leone Gazette* reported, “We have the satisfaction also to know, that the greater part of the African timber is imported for the most important purpose – that of building ships of the first class, as well for the royal navy as for the merchant service.”^22^ This was crucially important given the timber shortages facing the British navy by the end of the eighteenth century, after deforestation left them reliant on colonial sources and the loss of the thirteen American colonies reduced their access to North American supplies.^23^ In 1820, the *Sierra Leone Gazette* reported that “the timber trade, formed not above four years since, is now so far extended as to be not only one of the principal sources of supply to His Majesty’s dock yards, but also to be largely cultivated by private shipbuilders, and to have become, in a great degree, a regular branch of their trade among the principal timber merchants.”^24^

Freetown’s boosters in the press hoped that the local materials on offer in Sierra Leone would contribute to its attraction as a stopping point, and that the acquisition of adjacent territory would reduce competition from slave traders. As more merchants became involved in the timber trade, the colony’s commercial reach grew as new market entrants, including Liberated African settlers themselves, sought out new sources of cheap supply. In 1830, a British trader operating in Sierra Leone noted that timber merchants had spread out to the Melacourie River (in modern Guinea), where “The Timber is very fine, plentiful, and considerably cheaper than it is in the River Sierra Leone. . .the general opinion entertained of the River is clearly shewn by the fact that McCormack, Cole and Hamilton, Gabbidon and Campbell have all established factories there within the last few months.”^25^ Freetown’s merchants worked hard to corner the region’s timber trade throughout the early nineteenth century in order to provide a shipbuilding and repair material that would attract commerce to the colony.^26^

## Skilled labor

The presence of the Courts of Mixed Commission helped with the transfer of local knowledge about ship repair materials. When condemned slave ships were adjudicated through the Courts of Mixed Commission, the enslaved people on board were apprenticed out to the colony’s residents in order to provide them with the training that British philanthropists believed the Liberated Africans needed in order to become “productive” members of society.^27^ Those with existing skills were in particular demand in Freetown, and it is likely that, during the process of apprenticing and resettling the enslaved captives on a condemned ship, tradesmen would be particularly on the lookout for potential apprentices with promise, including those with existing experience in boatbuilding or ship repair.

Some 65,000 Liberated Africans were settled in Sierra Leone during the operation of the Court of Mixed Commission. In the 1831 census, there were 3,079 black colonial residents, 2,997 newly Liberated Africans, and 805 native strangers.^28^ These African migrants brought with them a variety of skills and experiences, which could be useful in generating knowledge of ship repair in Freetown that drew on boatbuilding, maintenance, and repair experience elsewhere in West Africa. Canoe-builders would have had experience repairing and taking care of wooden boats, including de-worming them, treating wood, caulking holes, adapting existing canoes for different environments as they aged, and beaching them to reduce rot.^29^ Some of these specific boat-repair skills, alongside general carpentry and ropemaking, were directly transferred to the repair of European wooden ships. Knowledge of West African boat maintenance would need to be combined with training or experience with European ships, and while some “native strangers” may have arrived with that experience, apprenticeship was another way of facilitating knowledge transfer. David Edmonds, an early settler in Freetown who came from a family of black loyalists from Nova Scotia, trained in England as a boat-builder and, after settling in Freetown, “within a few years he had several ship’s carpenters with him building small craft on the shore.”^30^

Liberated African apprentices were not the only Freetown residents to add to the rapid growth of the port’s reputation for ship repair. In the census, 504 Kru were reported to be living in the “Kroo Town” section of Freetown.^31^ Given that “Kru” was not an ethnic designation as such, but essentially a quasi-occupational one – the Kru were coastal mariners from the region stretching across Liberia and Côte d’Ivoire – it is highly likely that a significant portion of this population was proficient with boat construction and repair as well, since they had made their name as expert canoe-makers and auxiliary crewmembers on European naval, merchant, and whaling ships.^32^ Similarly, Robin Grafton, William Grafton, and John Snowball, based on Water Street and Cross Street Number 2, were all ship’s carpenters who were listed as “native strangers” in the colonial census, a designation that meant that they were not Liberated Africans or settlers, but African-born people who had moved into the colony from neighboring areas, bringing their skilled labor with them. The Graftons might have been Africans trained at places like the Isles de Los or Bunce Island, who brought with them specific knowledge of European ship carpentry, or canoe-builders who translated their experience of local materials into a new trade. In the eighteenth century, slaves hired out to the Royal Africa Company, for example, could be trained in European ship repair.^33^ The names of these three native strangers suggest that they had been apprentices in the colony, as they shared the names of earlier settlers.

There is also evidence of ship repair partnerships: Lewis Mitchell, a sail maker, and Nat Black, a ship’s carpenter, were based in the same property at 33 East Street. Ship’s carpenters also lived at the same addresses as sailors, other carpenters, and boatmen, which would enable knowledge spillover as pilots and mariners – as end-users of the vessels being repaired – contributed to discussions in social interactions. Clusters are linked to technological and entrepreneurial innovation in part through the concept of “knowledge spillover” as the growing cluster attracts a widening talent pool, as people from different firms share ideas when they move between firms, and as firms come up with unique solutions in order to draw customers away from rivals.^34^

By the late 1830s, Freetown’s ability to provide the kind of craftsmen needed on a trading voyage was common knowledge in Britain. A proposal seeking financing for a timber voyage noted that “a person should be engaged as Cooper and Carpenter for the voyage – or if that cannot be done a master Cooper may be had at Sierra Leone.”^35^ By the time of the 1831 Freetown Census, those who were listed specifically as ship’s carpenters made up nearly ten percent of the total number of carpenters in the colony. General carpenters made up roughly three percent of the total population of nearly 8,000 inhabitants. They were joined by rope makers, coopers, caulkers, blacksmiths, sail makers, and general merchants who specialized in ship chandlery. The ship repair industry was clustered on Back Street, Short Street, and Goderich Street, just past the markets at the harbor edge, and on the side streets just off Water Street, where many of the merchant warehouses were located. The cluster was well positioned to take advantage of the commercial traffic of the port not only to access customers, but also to access imported materials.

## Imported materials

The role of clusters has also been investigated with regard to the relationship between tacit knowledge such as the best substitute local materials, and global codified knowledge like how to repair copper bottoms or other wholly imported technologies.^36^ As has been seen in the contemporary literature on globalization and industry clusters, global pipelines – like the frequent port traffic from Europe and the United States, as well as the Court of Mixed Commission itself – that are combined with high levels of local innovation and “buzz” can create highly competitive clusters.^37^ The global knowledge that was transmitted by European-trained shipwrights like David Edmonds to his apprentices – including copper-bottom repair or sail repair – was incorporated with this local knowledge to make Freetown’s human capital as valuable as its material and environmental advantages. That local knowledge could include having the right contacts, for instance the auction agent for a condemned ship, to know when certain materials might be available. Some mercantile firms dominated the auction process for the condemned ships. Connections to these firms – which included Macaulay & Babington and Daniel Sutherland – could make it easier for shipwrights to source the correct global, imported supplies necessary for complicated repairs.

Freetown’s connections to global commerce, and its position as the site of the Courts of Mixed Commission after 1808, gave the colony access to imported ship repair materials in a volume that slave traders attempted to replicate without success. In Freetown itself, sails, ironwork, and planks were imported and kept in port to repair visiting ships. Early in the colony’s existence, Sierra Leone’s Governor Macaulay recorded an average of twenty-five visiting ships per year; by the late 1810s and 1820s, an average of forty visiting ships were recorded.^38^ This increasing volume of traffic both delivered necessary imports and generated a demand for repair work. In 1812, for instance, the colony imported 5 hundredweights of wrought copper, 5 hundredweights of iron nails, and 600 hundredweights of linen sail cloth from Britain.^39^ The colony’s mix of European and African settler merchants sourced goods from contacts in Liverpool, London, Aberdeen, Dover, Boston, and Bristol. The *Sierra Leone Gazette* reported in 1817 that the firm Macaulay and Babington had for sale “Copper Sheets and Nails, Naval stores of every sort: viz. Rope, Canvass, Anchors, Cables, Blocks, &c. &c. Paints and Oils.”^40^ These and other goods were imported on seven vessels from London and Aberdeen over the course of 1816, totaling nearly £16,500; in 1818 Macaulay and Babington received a further seven consignments for over £37,000.^41^ Other merchants advertised “ironmongery, paints, oils, and turpentine,” “carpenter’s tools,” “cables and anchors,” “sheet Lead and Copper Nails and spikes assorted.”^42^ The availability of these materials was appreciated by visiting ships, even if they complained about their expense. Martin Benson, captain of the Rhode Island Brig *Maria*, grumbled that he had spent $500 at Sierra Leone after “the loss of both anchors at Sierra Leone which were replaced from the Company’s Warehouse, the purchase of a new Cable” and the purchase of bread and pilot hire.^43^

These mercantile connections were important, but they were often matched by slave traders in the region with longstanding commercial connections in Europe and America. Imported materials for repairing ships were evidently available at the slave trading forts in the region, as records from some Rhode Island voyages show. The Rhode Island Schooner Olive Branch stopped at Bunce Island in 1803 to receive graving, caulking, and carpenter work on board for $11.50.^44^ The captain also paid a cooper $3 for iron hoops, rivets, and repair of two casks for ivory, and $12 for “sundry ship chandlery.”^45^ A schooner like the Olive Branch might be in more need of repairs after a long journey from Rhode Island – British traveler Anna Maria Falconbridge reported that it only took her eighteen days to reach Sierra Leone from Portsmouth, while a ship from Rhode Island could take six weeks.^46^ But the repairs were not intended to make the Olive Branch seaworthy for a return trip: it was sold in Africa at the end of the trading voyage. The schooner could then be used along the coast (in which case it would require future repairs to extend its life) or could be mined for materials to repair other ships.

As the slave trade was abolished and the power of the former slave trading companies began to dwindle, slave traders (or former slave traders) like Jan Neiser in Elmina further down the coast found themselves reliant on different, and ultimately more sporadic, sources of supply. Neiser, son of an official in the Dutch West India Company and an African woman named Manzang, wrote to Brown & Ives to:

Request your goodness to procure for me a good small size fast sailing schooner from 45 to 50 tonnage copper fasting in coppered to the bends well fitted out sails, rigging etc etc two good cables and three anchors, with two small boats 14 feet each and the under mentioned small cargo – to be sent out to me which I will paid the amount satisfactory.^47^

The existence of this market for whole ships shows how merchants along the West African coast combined access to local and global materials in order to conduct their own trade, keep their own vessels operating, replace old ships, and attract trade from ships in need of repair. In 1816, in response, Brown & Ives sent out three boats, “various sizes,” 118 oars, one large anchor, and five small anchors on the ship *Charlotte*.^48^ But ultimately, this was nothing like the volume of regular imports and ship chandlery from condemned slave ships that Freetown could offer, and increasingly those materials were accompanied by the growing cluster of Freetown’s skilled labor as well.

In Freetown, the former slave vessels condemned at the Court of Mixed Commission were manufactured originally in America and Europe, but in order to continue trading, they required refitting, repairs, and upkeep. The ships, formerly European, entered the hands of new African settler traders, further promoting ship repairing skills and interweaving the use of coastal and riverine crafts with larger seaworthy vessels. The Freetown Licenses Register records at least seventy-seven ship licenses and sufferances to ship issued between 1820 and 1842, reflecting the expansion of ship ownership among the colony’s black settlers and Liberated Africans.^49^ Almost all of the boats recorded were schooners, sloops, or cutters condemned in the Courts of Mixed Commission, although some went undesignated except by name. These licenses were issued to settlers including William Henry Savage, who was born in England to an English mother and African father, and had immigrated to the colony in 1808. Between September 1821 and July 1824, Savage received four ships’ licenses for the *Jane & Maria*, the *Swift*, and the *Nancy*.^50^ Savage was joined by William Carew, Robert Dougan, and Stephen Gabbidon, from among the black settlers, and George Nicol, a European who married into a prominent black settler family.^51^ By 1860, Freetown had thirty-eight registered ships.^52^ These locally owned vessels expanded commercial traffic and created a greater demand for ship repair services, which further expanded the tacit knowledge of ship repair among Freetown’s tradesmen as their experience of repair grew.

With 528 ships condemned by the court between 1819 and 1845 and sold to European merchant houses as well as groups of settlers who banded together to purchase them, the colony was clearly well stocked with the necessary materials for repairing both these locally owned and visiting ships.^53^ As a regular auctioneer and prize agent for the Court of Mixed Commission, the Freetown-based firm of Macaulay & Babington had access to a variety of ship chandlery from Brazilian, American, Spanish, Portuguese, British, and French ships captured by the Naval Squadron and brought for trial in Freetown.^54^ An advertisement by Macaulay & Babington in the *Sierra Leone Gazette* announced that the firm’s warehouse had for sale “The hull of the Ship Amelia, Sails, Masts, Yards, Running and Standing Rigging, Cables, Anchors, Boats and Water Casks” which were salvaged from the Court of Mixed Commission sales.^55^ Another advertisement, for “The good ship Esperanza, of the burthen of 209 tons or thereabouts. Foreign built, and condemned in the Vice-Admiralty Court of this colony, for a breach of the Slave Abolition Act” noted that the vessel was “new sheathed: well found, and excellently adapted for the timber or rice trade; *and is entitled to a British Register*.”^56^ Macaulay & Babington were not the only agents who were responsible for auctioning condemned slave ships, however, and so there was value to developing informal networks of information, which enhanced the role of the cluster in conveying an advantage to its participants. Tradesmen located near each other – or even at the same address – could learn quickly about upcoming auctions of scarce imported materials. Those with good working relationships with the expanding community of Liberated African coastal traders might have preferential information about local supplies of timber or oakum, or receive work when one purchased a newly condemned ship.

## Demand for repair work

As the colony sought to establish its competitive advantage as a port, the government itself also innovated in ways that assisted in attracting both customers and tradesmen. Prior to the establishment of the colony at Freetown, repairs, as well as the right to wood and water, were paid for with goods, not money.^57^ Establishing a currency-based, rather than trade-good based, system of payment was one of these innovations. As Governor of Sierra Leone before the handover of the colony to the British government, Zachary Macaulay commented that “The Cash paid the Company’s servants for Wages and the Settlers for Labour, returns into the Store and forms another very considerable branch of the issues of goods. To this may be added the goods issued for making or repairing buildings, or vessels, and for maintaining the Crews of Vessels.”^58^ A cash-based medium of exchange improved liquidity, as traders did not have to assort goods or rely on credit. From the perspective of the colony, it was also a boon as it ultimately tied all trade conducted in Sierra Leone dollars back to the colony. This cash-based monopoly was important for encouraging the transition to legitimate commerce and undermining the region’s slave traders. To buy any of the imported supplies on offer at the Sierra Leone Company Store, traders needed to exchange their legitimate products for cash: slaves were worthless for this trade. As Macaulay noted in 1797, “The Settlers many of whom have now boats carrying from two to Eight tons collect Rice, Camwood, and Live Stock, which they likewise dispose of for money with which to purchase a fresh assortment of Goods at the Store for trade.”^59^

After the colony was transferred to the British government in 1808, cash continued to be the preferred medium of exchange.^60^ In theory, the recipient of Freetown’s system of cash created a more streamlined payment method, where craftsmen could be paid directly, rather than through credit toward future goods purchased or trade goods sold. This cash-based system made the port attractive for craftsmen as well as for visiting ships because it gave them an additional option for payment, and it meant that ship’s carpenters like John Snowball would be remunerated directly, rather than through the mediation of someone like Jan Neiser, who “provided the services” to visiting ships – either by subcontracting to employees paid in goods or by using enslaved skilled laborers.

For example, in 1817, Captain Young of the Rhode Island trading vessel *Charlotte* required some basic repairs. As a small merchant ship with a minimal crew of 15–20 men at maximum, this was not a naval vessel equipped with a full crew that included expert tradesmen. Instead, like many minimally crewed “legitimate trade” vessels trading with Africa after the 1808 abolition of the slave trade, the captain relied on his knowledge of local ports to choose somewhere with skilled tradesmen who could undertake the repairs.^61^ In this case, Captain Young chose the Isles de Los, where the trained African associates of Samuel Samo, the Dutch-Surinamese, Africa-based trader, could provide “Carpenter, smith, and Cooper’s work” for $75 worth of goods in trade.^62^ A participant in the ongoing illegal slave trade, Samo’s ability to provide local repairs as well as skilled carpenters who could quickly construct the slave decks needed to transport enslaved people to the Americas gave his trading station a competitive advantage to the more lucrative coasting trade direct with African traders.^63^ But Samo was facing growing competition from Freetown, and increasingly, the financial services available at Freetown attracted both labor and customers to the port.

By 1820, further innovations among the merchant community in Freetown had expanded the repair cluster to include tertiary services. That year, the port of Freetown gained its own agent for Lloyd’s shipping insurance, Daniel Sutherland. Sutherland was one of the European merchants resident in the colony and an occasional auctioneer for the Courts of Mixed Commission. In his capacity as a broker, Sutherland ensured that Lloyd’s would underwrite repairs undertaken in the port for those who had insurance with the London brokerage. As Sutherland advertised, his role was to “prevent as far as in his power, any unfortunate dispute that might prevent an immediate settlement.”^64^ This upstream innovation in the cluster further expanded the services on offer for potential clients of the port. Sutherland had sought out the relationship with Lloyd’s in order to enhance his own business, with positive externalities for the port as a whole. Maritime insurance was an important innovation in long-distance trade that enabled the repair of transport technology.^65^ Having an insurance agent in a port, though, required entrepreneurial initiative spurred on by the kind of competition that takes place in clusters. It also again highlights the connections between global pipelines and local knowledge: Sutherland was linking up with a global insurer to pay for local repairs, reassuring potential customers that Freetown’s tradesmen’s technical knowledge was endorsed by the underwriters at Lloyd’s.

Both the colonial government and individual merchants innovated in the types of financial technologies available at Freetown. A cash-based economy helped the colony to stamp out the slave trade and gradually monopolize the legitimate commerce in the region. It attracted laborers as well as merchants, and it created the financial conditions that made the provision of British insurance underwriting possible. With the variety of primary, secondary, and tertiary economic activities offered by the repair cluster, ships involved in coastal African trade could easily see the benefit of coming to Freetown to trade, restock, and seek out any necessary repairs or maintenance.

## Conclusion

In the age of sail, the Sierra Leone ship repair cluster offered favorable environmental, material, and labor conditions. Knowledge transfer was facilitated by the presence of both West African and European materials: the local environment was as important as the global supply chain in enabling Freetown’s tradesmen in their repair of both locally owned and visiting ships. Certain innovations in the colony, including the purchase of well-known watering and wooding stops by the colonial government, the sale of Courts of Mixed Commission condemned ship parts, the apprenticeship of newly arrived Liberated Africans, and the use of cash for transactions in the colony, worked alongside the general expansion of commercial traffic in the port to attract labor into the trades as ship’s carpenters, rope makers, and sail makers, as well as shipping insurers.

The technical skills of these tradesmen were learned on the job either in Freetown, refitting and repairing the ships condemned by the Courts of Mixed Commission, or in other African contexts, repairing slave ships in the Isles de Los or Bunce Island, or mending, caulking, and retrofitting African canoes. This knowledge was combined with the environmental features of the region to create an economy that was well suited to the provision of ship repairs for long-distance trade, and one that the backers of the antislavery colony hoped would give them a competitive advantage over the slave trade. As slave voyages loading in the greater Sierra Leone region declined from a high of 364 ships in 1751–75, to 111 in 1826–50, to just 3 after 1851, Freetown’s commercial traffic boomed, growing from 25 ships in and out of the port in 1793, to 773 ships entered and cleared from the port in 1860, giving hope to those who had invested in the colony’s commercial approach to disrupting the slave trade.^66^

## Footnotes

1. British National Archives (hereafter BNA), CO 267/111, Census of Freetown and Liberated African Villages.

2. Michael Porter, “Clusters and the New Economics of Competition,” *Harvard Business Review* 76, 6 (1998): 77–90.

3. Christopher Fyfe, *A History of Sierra Leone* (Oxford: Oxford University Press, 1962); Bronwen Everill, *Abolition and Empire in Sierra Leone and Liberia* (Basingstoke: Palgrave Macmillan, 2013); Paul Lovejoy and Suzanne Schwarz (eds.), *Slavery, Abolition and the Transition to Colonialism in Sierra Leone* (Trenton: Africa World Press, 2015).

4. Padraic Scanlan, *Freedom’s Debtors* (New Haven: Yale University Press, 2017).

5. Edward Bold, *Merchant’s and Mariner’s African Guide* (London: J.W. Norie and Co, 1822), p.36; *The African Pilot, or Sailing Directions for the Western Coast of Africa, Part 1* (London: Hydrographic Office, 1856), p.78.

6. Jean Barbot, *A Description of the Coasts of North and South Guinea* (London, 1732), p.391.

7. Colleen Kriger, *Making Money* (Athens, OH: Ohio University Press, 2017), p.182.

8. Huntington Library, MSS MY 418, Journal of Zachary Macaulay, Folder 20, April 1, 1797.

9. BNA CO 271/2, *Sierra Leone Gazette*, January 26, 1822.

10. Bruce Mouser, “Trade and Politics in the Nunez and Pongo Rivers, 1790–1865” (PhD Diss, University of Wisconsin-Lacrosse, 1972), p.57.

11. BNA CO 270/1, *Sierra Leone Gazette*, July 18, 1818.

12. For example, Anna Maria Falconbridge, *Narrative of Two Voyages to the River Sierra Leone in 1791-2-3* (London, 1794), letter 3; BNA CO 271/1, *Sierra Leone Gazette*, February 26, 1820.

13. Patrick Manning, “Merchants, Porters and Canoemen in the Bight of Benin: Links in the West African Trade Network,” in Catherine Coquery-Vidrovitch and Paul Lovejoy (eds.), *The Workers of the African Trade* (Beverly Hills: SAGE, 1985), pp.51–72; John Thornton, *Warfare in Atlantic Africa* (London: Routledge, 1999); Jean-Pierre Chauveau, “Une histoire maritime africaine est-elle possible?” *Cahiers d’Études Africaines* 26, 101/102 (1986): 173–235; Peter Gutkind, “Trade and Labor in Early Precolonial African History: The Canoemen of Southern Ghana,” in Catherine Coquery-Vidtrovitch and Paul Lovejoy (eds.), *The Workers of the African Trade* (Beverly Hills: SAGE, 1985), pp.25–49; Robert Smith, “The Canoe in West African History,” *Journal of African History* 11, 4 (1970): 515–33, 520; Mariteuw Chimére Diaw, “Ethnogenesis, Mobility and Politics in the History of West African Canoe Fishermen,” in Inge Tvedten and Bjørn Hersoug (eds.), *Fishing for Development: Small-scale Fisheries in Africa* (Uppsala: Nordic Africa Institute, 1992), pp.54–69, 64.

14. Kevin Dawson, *Undercurrents of Power: Aquatic Culture in the African Diaspora* (Philadelphia: University of Pennsylvania, 2018), pp.114, 134–5; Peter C. W. Gutkind, “The Boatmen of Ghana: The Possibilities of a Pre-Colonial African Labor History,” in Michael Hanagan and Charles Stephenson (eds.), *Confrontation, Class Consciousness, and the Labor Process: Studies in Proletarian Class Formation* (Westport: Greenwood Press, 1986), pp.123–66; A. G. Hopkins, *An Economic History of West Africa*, 2nd ed. (London: Routledge, 2019).

15. Dawson, *Undercurrents of Power*, p.115 (note 14).

16. Ralph Austen and Daniel Headrick, “The Role of Technology in the African Past,” *African Studies Review* 26, 3–4 (1983): 163–84, 177, 179 n.21.

17. Stefan Tetzlaff, “Contested ‘automobility’: Peasants, townsfolk, and infrastructures of road transport in interwar central and western India (c. 1919–39),” *History of Science* 61 (2023): 77–101; Mary Draper, “Timbering and Turtling: The Maritime Hinterlands of Early Modern British Caribbean Cities,” *Early American Studies* 15, 4 (2017): 769–800; John McCusker and Russell Menard, *The Economy of British America, 1607–1789* (Chapel Hill: University of North Carolina Press, 1991); Sujit Sivasundaram, “Trading Knowledge: The East India Company’s Elephants in India and Britain,” *The Historical Journal* 48, 1 (2005): 27–63; Richard Drayton, “Maritime Networks and the Making of Knowledge,” in Richard Drayton and David Cannadine (eds.), *Empire, the Sea and Global History: Britain’s Maritime World, c. 1760–1840* (Basingstoke: Palgrave Macmillan, 2007), pp.72–82.

18. Kriger, *Making Money*, pp.182–3 (note 7).

19. BNA CO 267/9, *J. Thompson Commanding His Majesty’s Naval Forces in Africa*, March 9, 1785; Baring Archive HC17.60, Sierra Leone, December 31, 1839, from Peter Hartfield.

20. Fyfe, *History of Sierra Leone*, pp.125–6 (note 3); Huntington Library, MSS MY 369, August 16–September 7, 1830, Henry William Macaulay to Zachary Macaulay; BNA 270/8, Richard Bright’s Expedition to Moria in 1802; Bruce L. Mouser, “Continuing British Interest in Coastal Guinea-Conakry and Fuuta Jaloo Highlands (1750–1850),” *Cahiers d’études africaines* 172 (2003/4): 761–90.

21. BNA CO 267/111, *Census of Freetown and Liberated African Villages*.

22. BNA CO 270/1, *Sierra Leone Gazette*, September 9, 1820.

23. D. C. Dorward and A. I. Payne, “Deforestation, the Decline of the Horse, and the Spread of the Tsetse Fly and Trypanosomiasis (*nagana)* in Nineteenth Century Sierra Leone,” *Journal of African History* 16, 2 (1975): 239–56; M. M. Roche, “Forest Conservation for Royal Navy Timber Supplies in New Zealand,” *Mariner’s Mirror* 73, 3 (1987): 261–70, 261; Robert Albion, *Forests and Sea Power: The Timber Problem of the Royal Navy 1652–1862* (Cambridge, MA: Harvard University Press, 1926); Paul Bamford, *Forests and French Sea Power, 1660–1789* (Toronto: University of Toronto Press, 1956); Karl Apuhn, *A Forest on the Sea: Environmental Expertise in Renaissance Venice* (Baltimore: Johns Hopkins University Press, 2009); David M. Williams, “Merchanting in the First Half of the Nineteenth Century: The Liverpool Timber Trade,” *Business History* 8, 2 (1966): 103–121.

24. BNA CO 270/1, *Sierra Leone Gazette*, July 20, 1820.

25. Huntington Library, MSS MY 369, August 16–September 7, 1830, Henry William Macaulay to Zachary Macaulay.

26. Allen M. Howard, “The Relevance of Spatial Analysis for African Economic History: The Sierra Leone-Guinea System,” *The Journal of African History* 17, 3 (1976): 365–88; Bruce Mouser, “Trade, Coasters, and Conflict in the Rio Pongo from 1790 to 1808,” *The Journal of African History* 14, 1 (1973): 45–64; Huntington Library, MSS MY 645, Letters, November 17, 1827, Zachary Macaulay to Kenneth Macaulay; BNA WO 1/352, January 12, 1803, Questions proposed by Commodore Hallowell, with the Governor & Council’s answers.

27. Padraic Scanlan, “The Colonial Rebirth of British Anti-Slavery: The Liberated African Villages of Sierra Leone, 1815–1824,” *American Historical Review* 121, 4 (2016): 1085–1113; Richard Anderson, *Abolition in Sierra Leone* (Cambridge: Cambridge University Press, 2020), p.101.

28. Leslie Bethell, “The Mixed Commissions for the Suppression of the Transatlantic Slave Trade in the Nineteenth Century,” *Journal of African History* 7, 1 (1966): 79–93, 89; BNA CO 267/111, Census of Freetown and Liberated African Villages, p.257.

29. Dawson, *Undercurrents of Power*, pp.103–4 (note 14).

30. Fyfe, *Sierra Leone*, p.100 (note 3).

31. BNA CO 267/111, Census of Freetown and Liberated African Villages.

32. Dawson, *Undercurrents of Power*, p.58 (note 14); James Hornell, “The Kru Canoes of Sierra Leone,” *The Mariner’s Mirror: the Journal of the Society for Nautical Research* 15 (1929): 233–7; George E. Brooks, Jr., *The Kru Mariner in the Nineteenth Century* (Newark: Liberian Studies Association, 1972); George Brooks, *Eurafricans in Western Africa* (Athens, OH: Ohio University Press, 2003); Robert Burroughs, “‘The True Sailors of Western Africa’: Kru Seafaring Identity in British Travellers’ Accounts of the 1830s and 1840s,” *Journal for Maritime Research* 11, 1 (2009): 51–67; W. Jeffrey Bolster, *Black Jacks* (Cambridge, MA: Harvard University Press, 2009); Jane Martin, “Krumen ‘Down the Coast’: Liberian Migrants on the West African Coast in the 19th and early 20th centuries,” *International Journal of African Historical Studies* 18, 3 (1985): 401–23; Diane Frost, “Ethnic Identity, Transience and Settlement: The Kru in Liverpool since the Late Nineteenth Century,” *Immigrants and Minorities* 12, 3 (1993): 88–106.

33. Rebecca Shumway, *The Fante and the Transatlantic Slave Trade* (Rochester: Rochester University Press, 2011), p.63; Randy Sparks, *Where the Negroes Are Masters* (Cambridge, MA: Harvard University Press, 2014); Ty Reese, “Facilitating the Slave Trade: Company Slaves at Cape Coast Castle, 1750–1807,” *Slavery & Abolition* 31, 3 (2010): 363–77.

34. For examples of the application of the idea of an industrial cluster in African development, see S. A. Rosenfeld, “Bringing Business Clusters into the Mainstream of Economic Development,” *European Planning Studies* 5, 1 (1997): 3–23; Banji Oyelaran-Oyeyinka, “Knowledge Networks and Technological Capabilities in the African Manufacturing Cluster,” *Science, Technology and Society* 8, 1 (2003): 1–23; Nonso Obikili, “Social Capital and Human Capital in the Colonies: A Study of Cocoa Farmers in Western Nigeria,” *Economic History of Developing Regions* 30, 1 (2015): 1–22.

35. Baring Archive HC17.60, Sierra Leone, December 31, 1839 from Peter Hartfield to Messrs Baring Brothers and Co.

36. Harald Bathelt, Anders Malmberg, and Peter Maskell, “Clusters and Knowledge: Local Buzz, Global Pipelines and the Process of Knowledge Creation,” *Progress in Human Geography* 28, 1 (2004): 31–56.

37. Bathelt, Malmberg, and Maskell, “Clusters and Knowledge” (note 36); Anders Malmberg and Dominic Power, “(How) Do (Firms in) Clusters Create Knowledge?” *Industry and Innovation* 12, 4 (2006): 409–31; Bjorn Asheim and Arne Isakson, “Regional Innovation Systems: The Integration of Local ‘Sticky’ and Global ‘Ubiquitous’ Knowledge,” *Journal of Technology Transfer* 27 (2002): 77–86.

38. Huntington Library, MSS MY 418, Macaulay’s Journal, between 1793 and 1799; BNA CO 267/91 A 30, An Account of the Imports of the Colony of Sierra Leone in the period from January 1817 to December 1825.

39. BNA CUST 8, 1812. Most years did not disaggregate African ports; 1812 is an exception.

40. BNA CO 270/1, *Sierra Leone Gazette*, August 2, 1817.

41. BNA CO 267/91, A 30, An Account of the Imports of the Colony of Sierra Leone in the period from January 1817 to December 1825.

42. BNA CO 271/2, *Sierra Leone Gazette*, March 3, 1821; November 22, 1817.

43. John Carter Brown Library (hereafter JCB) BFBR B.591 F.5, Brig Maria Cape Coast Castle, October 1, 1800, Benson to B&I.

44. JCB BFBR, Vessels Schooner Olive Branch, Benson Trade Book, September 20, 1802–March 16, 1803. The price in dollars reflects the cost in Sierra Leone currency.

45. JCB BFBR B.611, Olive Branch Trade Book, Disbursements Bance Island.

46. Falconbridge, *Two Voyages in Sierra Leone*, Letter 3 (note 12).

47. JCB BFBR B629 F.1, Elmina, October 10, 1817, Jan Neiser at Elmina to B&I; J. T. Lever, “Mulatto Influence on the Gold Coast in the Early Nineteenth Century: Jan Neiser of Elmina,” *African Historical Studies* 3, 2 (1970): 253–61.

48. JCB BFBR B.523 F.8, Shipped from Brown & Ives on the Ship Charlotte, Gideon Young Master, September 26, 1816.

49. Sierra Leone National Archives (hereafter SLNA), Fourah Bay College, Freetown, License Book, 1820–42.

50. SLNA License Book, index.

51. SLNA License Book, index. On Nicol, see Fyfe, *Sierra Leone*, p.103 (note 3).

52. J. Forbes Munro, “African Shipping: Reflections on the Maritime History of Africa South of the Sahara, 1800–1914,” *International Journal of Maritime History* 2, 2 (1990): 163–82, 170–71.

53. Bethell, “Mixed Commissions,” p.84 (note 28).

54. Scanlan, *Freedom’s Debtors*, p.175 (note 4).

55. BNA CO 270/1, *Sierra Leone Gazette*, August 23, 1817.

56. BNA CO 270/1, *Sierra Leone Gazette*, August 2, 1817.

57. Paul Hair (ed.), *Barbot on Guinea: The Writings of Jean Barbot on West Africa, 1678–1712 volume 1* (London: Hakluyt Society, 1992), pp.184-5. See pages 219-20 for a list of the types of goods in demand.

58. Huntington Library, MSS MY418, Folder 21, June 7, 1797: Remarks on the Health, Trade, Cultivation, and Civilization of Sierra Leone.

59. Ibid.

60. Sophie Mew, “Trials, Blunders and Profits: The Changing Contexts of Currencies in Sierra Leone,” *Journal of Imperial and Commonwealth History* 44, 2 (2016): 195–213.

61. Non-slave trade vessels did not require large crews to maintain order in the same way that slaving ships did.

62. JCB BFBR B523 F.10, Ship Charlotte to Africa 1816–17, June 22, 1817, Samuel Samo bill to Ship Charlotte; British and Foreign State Papers, Volume 11 (1843), 433; Bruce Mouser, “The Trial of Samuel Samo and the Trading Syndicates of the Rio Pongo, 1797 to 1812,” *The International Journal of African Historical Studies* 46, 3 (2013): 423–41.

63. Some ships brought their own carpenters with them to transform ships along the route and deconstruct them after Caribbean departures in order to arrive back in Europe without evidence of a slaving voyage. See Jessica A. Glickman, “A War at the Heart of Man: The Structure and Construction of Ships Bound for Africa,” (PhD Diss., University of Rhode Island, 2015), p.44; Stephen Behrendt, “Markets, Transaction Cycles, and Profits: Merchant Decision Making in the British Slave Trade,” *The William and Mary Quarterly* 58, 1 (2001): 171–204, 176.

64. BNA CO 270/1, *Sierra Leone Gazette*, August 2, 1817.

65. See Mary Brazelton and Daniel Margocsy, “From Hansa to Lufthansa: Transportation Technologies and the Mobility of Knowledge,” *History of Science* (Forthcoming).

66. Transatlantic Slave Trade Database, https://slavevoyages.org/voyages/sg1yCgpI; Huntington Library MSS MY 418, Macaulay’s Journal; Sierra Leone Blue Books 1860, British Foreign and Commonwealth Office, Si09-37, 262–5. 1860: 383 entered and 390 cleared (10 April 2020).

